# ESBL-Producing *Escherichia coli* and *Klebsiella pneumoniae* Exhibit Divergent Paths During In-Human Evolution Towards Carbapenem Resistance

**DOI:** 10.3390/microorganisms13061387

**Published:** 2025-06-14

**Authors:** Michelle Chioma Kalu, Akanksha Acharya, Peter Jorth, Annie Wong-Beringer

**Affiliations:** 1 Titus Family Department of Clinical Pharmacy, Alfred E. Mann School of Pharmacy and Pharmaceutical Sciences, University of Southern California, Los Angeles, CA 90033, USA; 2 Department of Pathology and Laboratory Medicine, Cedars-Sinai Medical Center, Los Angeles, CA 90048, USA

**Keywords:** carbapenem resistance, ESBL-producing *Enterobacterales*, genetic evolution, CRISPR-Cas

## Abstract

Treatment of infections caused by ESBL-producing *Escherichia coli* (EC) and *Klebsiella pneumoniae* (KP) with carbapenem antibiotics can lead to the development of carbapenem resistance over time through the acquisition of porin mutations and plasmids bearing *blaKPC*. However, the impact of genetic background and the presence of CRISPR-Cas systems on the evolutionary path towards carbapenem resistance in EC and KP has yet to be investigated. The in-human evolution following repeated carbapenem treatment among ESBL-producing *Escherichia coli* (EC) and *Klebsiella pneumoniae* (KP) clinical pairs (n = 45 pairs) was examined to determine the relationship between strain genetic background (MLST, CRISPR-Cas) and the evolved genetic mutations related to resistance, virulence, and metabolism by whole genome sequencing. ST131 and ST258 were predominant among seven distinct STs in EC (70%, 19/27) and 11 STs in KP (33%, 6/18), respectively. Complete CRISPR-Cas systems were present in 22% EC (6/27) and 27.8% (5/18) KP pairs, but none in strains belonging to ST131 or ST258; partial loss of CRISPR-Cas was associated with increased carbapenem resistance. Porin, virulence, and metabolism-related genetic mutations were present on the chromosome in both the EC and KP evolved strains, but their presence was differentially associated with the CRISPR-Cas system. Future research on the role of antibiotic exposure in the species-specific resistance evolution of the *Enterobacterales* could guide antimicrobial stewardship efforts.

## 1. Introduction

Carbapenem-resistant Enterobacterales (CRE) are a global concern and deemed an urgent threat by the Centers for Disease Control and Prevention, with significant increases in rates of hospital-onset CRE infections [1,2]. Prior infection with extended-spectrum beta-lactamase (ESBL)-producing *Enterobacterales* has been shown to be a significant risk factor for the subsequent development of carbapenem-resistant infections (CR) [3,4]. The carbapenem agents are considered the treatment of choice for serious infections caused by ESBL-*Enterobacterales* regardless of whether *Escherichia coli* (EC) or *Klebsiella pneumoniae* (KP) are involved [5,6]. Exposure to carbapenem therapy facilitates the acquisition of carbapenemase encoded on multidrug resistance plasmids or the acquisition of mutations in genes encoding for the outer membrane porins as well as key enzymes involved in metabolism to increase resistance and adaptation for survival [7,8]. Prior exposure to carbapenem therapy may also support the development of heteroresistance in which a subpopulation of carbapenem-susceptible strains displays increased resistance to carbapenem agents [3,9,10]. While EC are more prevalent than KP strains among ESBL-producing *Enterobacterales*, carbapenem resistance was observed to be more prevalent among KP strains belonging to ST258. KP strains developed resistance either through the acquisition of mutations in porin genes and/or through the acquisition of carbapenemase-containing plasmids, while EC strains appeared to be more likely to exhibit carbapenem-heteroresistant (cHR) phenotype [11,12]. While carbapenem exposure is a known driver of the development of carbapenem resistance, a strain-dependent difference in the evolutionary path towards carbapenem resistance in EC and KP likely exists but has yet to be investigated. One potential contributing factor could be the presence of CRISPR-Cas systems in the genomes of many EC strains but not in the epidemic KP strains belonging to ST258 as the presence of CRISPR-Cas systems has been shown to impact the development of multidrug resistance phenotypes [13].

CRISPR-Cas systems serve as one of many defense mechanisms employed by bacterial organisms to defend against foreign genetic material such as phages and plasmids [14]. Multiple studies have attributed the increased acquisition of carbapenemase-encoding plasmids to the lack of CRISPR-Cas systems in ST258 strains of KP [15,16,17]. Moreover, carbapenem exposure has been shown to directly influence the expression of CRISPR-Cas systems in KP [18]. Thus, we hypothesized that the presence of CRISPR-Cas systems, in addition to genetic background, supports the differential evolution of carbapenem resistance in ESBL-producing EC and KP strains. We have curated a unique collection of ESBL-EC and -KP strains from patients with repeated colonization or infection to track the evolution of carbapenem resistance over time by (1) determining the distribution of CRISPR-Cas systems and genetic backgrounds in ESBL-*Enterobacterales* and (2) comparing the prevalent genetic mutations related to resistance, virulence, and metabolism in evolved strains, exhibiting different carbapenem resistance phenotypes. Our results showed that while most ESBL-*Enterobacterales* strains carried CRISPR-Cas systems irrespective of EC or KP, acquisition of porin mutations associated with carbapenem resistance mostly occurred in CRISPR-Cas positive EC strains compared to KP strains, while KP evolved strains that acquired the plasmid containing *blaKPC* (gene encoding *K. pneumoniae* carbapenemase enzyme), mediating carbapenem resistance carried no CRISPR-Cas systems. Additionally, among CRISPR-Cas negative strains, acquisition of mutations in genes related to virulence was observed in both EC and KP while mutations in genes related to metabolism were mostly observed in evolved KP strains. This study highlights the influence of bacterial genetic background on the divergent evolution between ESBL-producing EC and KP clinical strains following repeated carbapenem exposure, with implications for the differential use of treatment strategies for ESBL-Enterobacterales infections depending on the pathogen.

## 2. Materials and Methods

### 2.1. Study Isolates

A total of 45 patients with repeated infections or colonization during different hospital admissions were selected for this study. The study was conducted on patients hospitalized from 2014 to 2021 under an IRB-approved protocol #HS-17-00943. Informed consent was waived as no interventions were made. Inclusion criteria were that the index strain was documented to exhibit ESBL-producing phenotype on clinical microbiology laboratory records, the evolved strain causing infection or colonization occurred within two years after the index infection based on positive culture, and that both the index and subsequent “evolved” strains were cryopreserved. The study included 30 female and 15 male patients. Strains were selected to represent various infection sites, including urine, blood, wounds, trachea, and sputum. Carbapenem heteroresistance was determined by population analysis profile (PAP) assay in a previous study [19]. All strains were grown at 37 °C from frozen stocks using CHROMagar Orientation media (CHROMagar, Saint-Denis, France), the colonies were then inoculated in LB broth and incubated at 37 °C with shaking overnight. Broth microdilution susceptibility testing to meropenem (Sigma, St. Louis, MO, USA) was performed for select pairs of isolates with porin mutations in triplicate in cation-adjusted Mueller–Hinton broth (Sigma) per CLSI guidelines [20].

### 2.2. DNA Extraction

Whole genome DNA was extracted from all study strains using the QIAmp DNA Extraction Micro Kit (Qiagen, Hilden, Germany), with slight modifications to the protocol. Briefly, overnight bacterial inoculum was treated with RNase A (Qiagen) and proteinase K (Qiagen), incubated for 2 h at 56 °C, then extracted per manufacturer’s protocol. Extracted DNA from all the samples were checked for purity and concentration using the NanoDrop spectrophotometer (ThermoFisher, Waltham, MA, USA).

### 2.3. Rapid Amplification of Polymorphic DNA (RAPD) Assay

RAPD assay was used to screen for clonal relatedness between the index and evolved strains of each clinical pair. PCR reactions were performed using 250 ng of extracted DNA and GoTaq^®^ Green Master Mix 2x (Promega, Madison, WI, USA) in 25 uL volume per reaction. The PCR amplification was repeated for 50 cycles using an annealing temperature of 34 °C. The primers used were HLWL 74 (5′ ACGTATCTGC 3′), 640 (5′ CGTGGGGCCT 3′), and AP4 (5′ TCACGATGCA 3′) at 1.0 μM concentration. All PCR products were loaded on 1.5% agarose gel with 10 μg/mL ethidium bromide in 1× TAE buffer for gel electrophoresis and run for 1 h at 100 V to visualize banding pattern.

### 2.4. Whole Genome Sequencing and Analysis

Extracted DNA was loaded onto the 1.5% agarose gel with ethidium bromide in 1x TAE Buffer and electrophoresed for 30 min at 100 V to confirm a single band without fragmentation for ease of sequencing and library preparation. DNA samples were then sent to SeqCenter and Genewiz from Azenta Life Sciences for library preparation and whole genome sequencing at 2 × 150 bp read length using Illumina MiSeq (San Diego, CA, USA). All the genomes were assembled and annotated using the Comprehensive Genome Analysis service using default settings from the Bacterial and Viral Bioinformatics Resource Center (BV-BRC, version 3.44.4) [21,22]. The presence of any CRISPR-Cas system was determined using CRISPRCas MetaFinder (version 1.1.2) [23]. Presence of a complete CRISPR-Cas systems was defined as presence of all protein-encoding genes as well as CRISPR array in the genome. Partial loss was defined as deletion of either Cas protein-encoding genes or CRISPR arrays. Assembled genomes were further analyzed for mutations by comparison to a reference genome with the same MLST using *breseq* (version 0.38.2) [24]. Reference genomes were obtained from the public NIH NCBI Nucleotide Database, either through manual searches for FASTA files corresponding to the specific MLST or by utilizing the Similar Genome Finder tool on BV-BRC (version 3.44.4). Gene alignments were performed using MAFFT aligner on default settings and converted to protein sequences in Geneious (Geneious Prime 2024.0.4, GraphPad, Boston, MA, USA). To identify plasmid replicons in EC and KP sequences, a custom BLAST (version 2.12.0) was created in Geneious Prime using the most recent PlasmidFinder database (updated February 2017) [25]. BLAST results were confirmed using a pairwise identity threshold of 95%.

### 2.5. Statistical Analysis

All statistical analysis including Fisher’s exact test was performed using Prism 10 (GraphPad, version 10.4.1).

## 3. Results

### 3.1. ESBL Clinical Strains from Diverse Genetic Backgrounds Evolved Different Carbapenem-Resistant Phenotypes

Study strains were identified from microbiology records and selected for the study if all of the following criteria were met: culture positive for the evolved strain separated by at least one month and no more than two years after the index strain, both index and evolved strains from the same patients were cryopreserved, and the index and evolved strains were clonally related as determined by whole genome sequencing.

**Figure 1:**
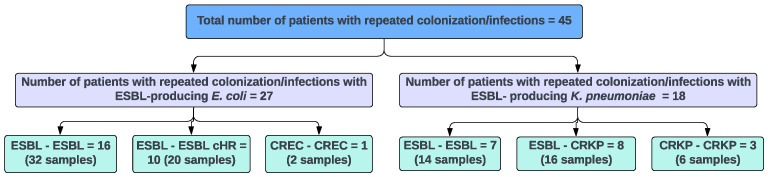
Resistance phenotypes of EC and KP pairs isolated from patients with repeated colonization or infection.

**Figure 2:**
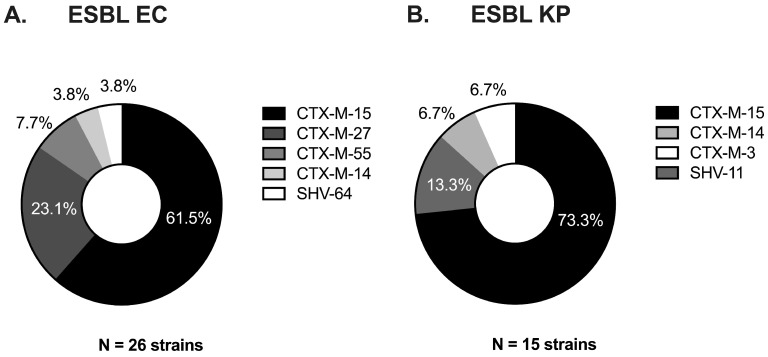
ESBL gene variants observed across index strains of (**A**) ESBL EC and (**B**) ESBL KP. Genomic reads from whole genome sequencing were assembled and analyzed using Comprehensive Genome Analysis by BV-BRC.

A total of 90 index and subsequently evolved EC and KP strains that caused repeated colonization or infection in 45 patients were selected for the study. The majority of the isolates were obtained from urine (73%, 66/90), followed by blood (10%, 9/90), respiratory source (10%, 9/90), and wound (7%, 6/90). Figure 1 depicts the resistance phenotypes exhibited by the 45 pairs of study strains; 10 of which comprised ESBL-EC evolved to carbapenem-heteroresistant EC and 8 ESBL-KP evolved to carbapenem-resistant *K. pneumoniae* (CRKP). Additional clinical pairs of ESBL-EC (n = 16), ESBL-KP (n = 7), CREC (n = 1), and CRKP (n = 3) with unchanged carbapenem susceptibility between the index and evolved strains were included as controls for comparison. The majority of index ESBL strains of both EC and KP pairs contained the ESBL-encoding gene *blaCTX-M-15* (Figure 2; 62%, 16/26 and 73%, 11/15, respectively). The remaining strains carried variants of *blaCTX-M* and/or *blaSHV* genes.

**Figure 3:**
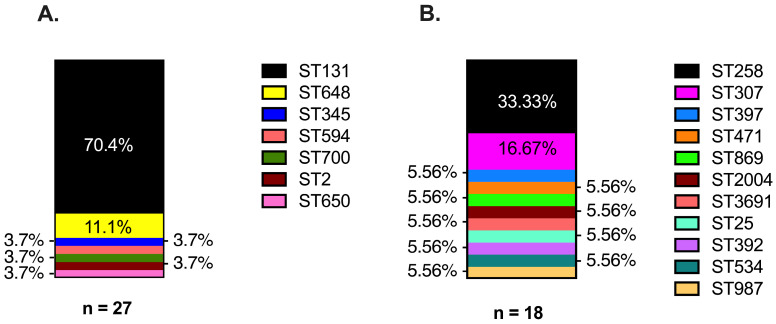
Multiple Locus Sequence Types (MLSTs) observed across evolved strains of (**A**) EC and (**B**) KP. Genomic reads from whole genome sequencing were assembled and annotated using Comprehensive Genome Analysis by BV-BRC. MLST assigned to strains as part of the analysis performed by the Comprehensive Genome Analysis program.

Clonal relatedness of the strain pairs was screened by the RAPD assay and confirmed along with strain sequence type by MLST analysis of the whole genome sequencing (WGS) results. Our results confirmed that the index and evolved strains of all the patients were clonally related and that a majority of the study strains belonged to the globally recognized ST131 and ST258 for EC and KP, respectively. Notably, KP isolates included 11 distinct MLSTs, while the EC isolates comprised seven distinct MLSTs (Figure 3). ST131 (70%, 19/27 pairs) followed by ST648 (11%, 3/27 pairs) were the most common MLSTs found among the EC strains. ST131 was the predominant type among the EC strains irrespective of the carbapenem susceptibility phenotype of the evolved strains: 69% (11/16) ESBL-ESBL EC; 70% (7/10) ESBL-cHR EC pairs, and the single CREC-CREC pair. Among the 18 pairs of KP strains, ST258 was most prevalent (33%, 6/18 pairs), which represented 38% (3/8) of the ESBL-CRKP pairs and all of the CRKP-CRKP pairs. On the other hand, three pairs of KP strains belonged to ST307; two of which were among the seven ESBL-ESBL KP pairs (28%).

### 3.2. Strains with Absent or Progressive Loss of CRISPR-Cas Systems Support Plasmid-Bearing blaKPC

We analyzed the assembled contigs for the presence of CRISPR-Cas systems in relation to the evolved carbapenem-resistant phenotypes. Complete CRISPR-Cas systems were similarly prevalent among EC pairs of ESBL-EC strains compared to ESBL KP pairs irrespective of evolved carbapenem-resistant phenotypes (22%, 6/27, and 27.8% 5/18, respectively). A total of 10 of the 45 index strains contained complete CRISPR-Cas systems consisting of the Type I-E subtype, except for one KP strain in the ESBL-ESBL KP group which had CRISPR-Cas Type I-E*. Of note, none of the strains belonging to ST131 or ST258 genetic background contained CRISPR-Cas systems. Similarly, none of the EC or KP pairs with carbapenem resistance for both index and subsequent strains (CREC-CREC; CRKP-CRKP) carried complete CRISPR-Cas systems. The single CREC pair was *blaKPC* positive carrying a *blaKPC-2* gene, while the majority of CRKP strains were *blaKPC* positive with either *blaKPC-2* or *blaKPC-3* genes (71%, 10/14). One CRKP strain carried a *blaOXA-232* gene, classified as a class D carbapenemase [26]. Of the four strains classified as CRKP but negative for *blaKPC*, two carried complete CRISPR-Cas systems. Three clinical pairs exhibited partial loss of CRISPR-Cas systems in the evolved strains (Table 1). Index strains from two ESBL-ESBL EC pairs (ST131) containing only the *cas3* gene had deletion of the gene in the evolved strain exhibiting carbapenem heteroresistant phenotype and another without change in carbapenem susceptibility; one pair of ESBL-KP index strain containing only the *cas5* gene had loss of the gene in the evolved CRKP strain.

The blaKPC gene has been found primarily on plasmids with IncR, IncFII, and IncFIB replicons [27]. To identify if there was a relationship between the presence of CRISPR-Cas systems and plasmid types, a custom BLAST was performed using the PlasmidFinder database to identify plasmid replicons within the sequences of evolved EC and KP strains. The IncFIB replicon was the most prevalent plasmid replicon in both evolved EC and KP strains (77.8%, 21/27, and 77.8%, 14/18, respectively). The presence of IncFIB replicon did not differ between CRISPR-positive (5/6, 80.0% and 4/5, 83.3%) and CRISPR-negative strains (10/13, 76.9%, and 16/21, 76.2%) in evolved EC and KP strains, respectively. Interestingly, these results suggest that the CRISPR-Cas system in *E. coli* and *K. pneumoniae* does not influence the acquisition of plasmid types including those likely to carry the *blaKPC* gene.

### 3.3. Acquisition of Porin Mutations Differs Between ESBL EC and KP Strains by CRISPR-Cas Background

Analysis was performed on the sequenced contigs using *breseq* to identify mutations acquired by the evolved strains that may support differential evolution between ESBL-EC and ESBL-KP towards carbapenem resistance (Supplemental Data S1). Among EC strains, non-synonymous mutations in porin genes *ompC* and *ompF* as well as *ompR* were identified in 50% (3/6) of CRISPR-Cas positive pairs compared to none (0/21) of the CRISPR-Cas negative pairs (Table 2). When compared by evolved phenotype, two of the ESBL-EC pairs with carbapenem-heteroresistant phenotype in the evolved strain showed mutations in *ompF* and *ompR*, respectively. In one of the pairs where carbapenem susceptibility was maintained in the evolved strain, the evolved strain acquired mutations in *ompC*. No porin mutations were observed in the single pair of CRISPR-Cas negative *blaKPC* positive carbapenem-resistant EC strains (Table 2).

In comparison to EC strains, a higher proportion of the evolved KP strains acquired nonsynonymous mutations in porin-encoding genes (*ompK35* (*ompF*) and *ompK36* (*ompC*)), though not statistically significant (11.1%, 3/27 vs. 22.2% 4/18, Fisher’s exact *p* = 0.412). Unlike EC strains, the proportion of evolved KP strains with porin mutations did not differ by the presence of CRISPR-Cas system (20%, 1/5 CRISPR-Cas positive vs. 23%, 3/13 CRISPR-Cas negative strains) (Table 3).

**Figure 4:**
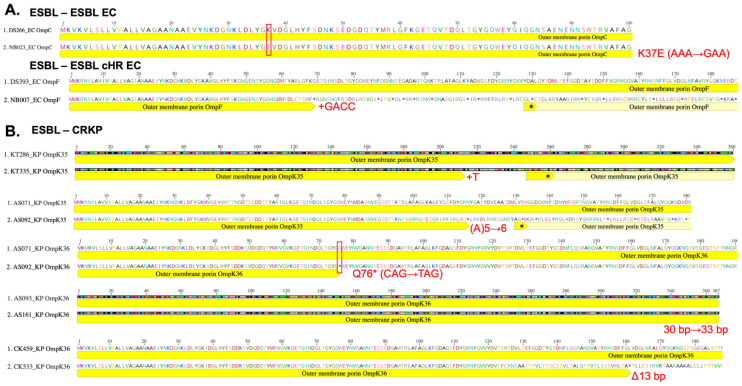
Protein sequence alignments of porins between index and evolved (**A**) EC and (**B**) KP pairs with identified mutations in the coding region of the genes. MAFFT alignments performed using Geneious software. Specific mutations identified in the evolved strain are noted in red text. Red boxes highlight single polymorphisms. (*) Denotes a stop codon in the sequence.

Among the ESBL-KP pairs with evolved CRKP, 50% (4/8) also carried porin mutations; all four strains were blaKPC negative. Of the four pairs with porin mutations, two ESBL-CRKP pairs were CRISPR-Cas negative where one acquired a base pair insertion in *ompK35* and the other acquired a base pair insertion in *ompK35* and a nonsynonymous mutation, resulting in a frameshift mutation and early termination in *ompK36*. For the other two pairs which were CRISPR-Cas positive, both evolved strains acquired a 13 base pair deletion in porin gene *ompK36* (Figure 4).

ESBL-CRKP pairs with acquired porin mutations resulting in termination of the protein demonstrated increased meropenem MICs of at least four-fold. The ESBL-cHR EC pair with an acquired porin mutation exhibited a two-fold increase in meropenem MIC, emphasizing the difference in carbapenem resistance phenotypes (Table 4). Interestingly, this increase in meropenem MICs occurred without the presence of *blaKPC* genes in evolved CRKP strains and regardless of the presence of CRISPR-Cas systems. Taken together, porin mutations appear to represent a path towards evolving carbapenem heteroresistance in CRISPR-Cas positive EC strains, while KP strains may be less stringent in acquiring non-synonymous porin mutations to support the development of low levels of carbapenem resistance in CRISPR-Cas positive strains as well as high-level carbapenem resistance in combination with plasmid *blaKPC* acquisition in the absence of CRISPR-Cas systems.

### 3.4. Acquisition of Virulence Gene Mutations Favored CRISPR-Cas Negative EC and KP Strains

Among our clinical pairs of ESBL-EC and KP strains, the acquisition of non-synonymous mutations in several virulence genes involved in LPS and capsule synthesis, siderophore production and uptake, and pilus motility was observed and at similar frequencies between the evolved EC and KP strains (37%, 10/27 EC vs. 22%, 4/18 KP) (Table 2 and Table 3). Notably, two evolved EC strains acquired mutations in *agn43*, producing antigen 43, known to be involved in biofilm formation [28]. The majority of the mutations occurred only in the evolved strains of EC and KP that were CRISPR-Cas negative and were found only on the chromosome. Interestingly, virulence gene-related mutations were observed in EC strains irrespective of evolved carbapenem resistance phenotype but were only observed among KP strains bearing blaKPC.

### 3.5. Acquisition of Mutations in Metabolism-Related Genes Favors CRISPR-Cas Negative KP but Not EC Strains

Evolved EC and KP strains appeared to acquire mutations in different metabolism-related genes (Table 2 and Table 3). Mutations were observed on the chromosome of EC strains (ESBL-ESBL or ESBL-ESBL cHR) irrespective of CRISPR-Cas background and carbapenem susceptibility, which included *hycE*, *frdA*, and *aaeB* encoding proteins involved in the tricarboxylic cycle and metabolic waste disposal, *cydA* encoding cytochrome ubiquinol oxidase subunit I, *lacY* encoding lactate permease, and *dtpA* and *dtpC* encoding proteins involved in the transport of dipeptides and tripeptides. The single CREC pair did not contain mutations in genes related to metabolism in the evolved strain. On the other hand, mutations in genes related to glucose and nitrate metabolism, stress response, and the electron transport chain were only observed in CRISPR-negative evolved KP strains that were also CRKP bearing plasmid-*blaKPC*; none were observed in ESBL-KP strains where carbapenem susceptibility remained unchanged.

## 4. Discussion

The rise in infections caused by carbapenem-resistant *Enterobacterales* has prompted further investigations into the evolution of carbapenem resistance in ESBL-producing *E. coli* and *K. pneumoniae*. The higher prevalence of CRKP compared to CREC strains, despite a greater prevalence of ESBL-EC compared to ESBL-KP, raised questions about whether EC and KP differentially evolve resistance under repeated carbapenem exposure. We hypothesized that the difference in the strain genetic background with or without the presence of CRISPR-Cas systems supports the differential evolution of carbapenem resistance in ESBL-EC and KP strains. Forty-five pairs of isolates were selected from patients who experienced repeated colonization or infections. This unique collection of strains afforded us the opportunity to investigate the in-human evolution of carbapenem resistance in EC and KP strains as well as the role of CRISPR-Cas systems in the acquisition of plasmids supporting the development of carbapenem resistance. Whole genome sequencing was performed to confirm the clonal relatedness of each pair of index and evolved study strains and to identify the presence of CRISPR-Cas systems, plasmid-bearing *blaKPC* and ESBL genes, and mutations in genes related to resistance, virulence, and metabolism in the evolved strains. Our strain collection was most frequently isolated from the urine (73%), followed by blood (10%) and respiratory (10%) sources. Several studies have highlighted the bladder as a potential reservoir for evolution of resistance and virulence characteristics due to the ease of forming stationary biofilm communities where genetic material can be shared and the strains can evolve undisturbed by antibiotics or the immune system [8,29,30].

With respect to the strain genetic background, the majority of ESBL-cHR EC pairs as well as the CREC-CREC pair belonged to ST131, while most of the carbapenem-resistant KP pairs belonged to ST258; the latter is the product of genetic rearrangement and the merging of two genetic clones, allowing for more fluid changes in its genetic background [31]. Consistent with previously the published literature, none of these epidemic strains carried CRISPR-Cas systems, indicating that CRISPR-Cas systems do not co-exist with strains harboring plasmid-bearing *blaKPC* [32]. We also observed that there was no association between the presence of plasmid replicon IncFIB, which was most common in evolved EC and KP strains, and the presence of CRISPR-Cas. Of interest, partial loss of CRISPR-Cas systems may support strains as they evolved toward carbapenem-resistant phenotypes in both EC and KP strains. Other studies have also observed the impact of the biological environment on CRISPR-Cas expression. For example, increased glucose in the environment has been shown to induce cas gene expression in *E. coli* strains, and in *Salmonella* bacteremia cas3 expression was upregulated [33,34]. Future studies will investigate the impact of antibiotic exposure on the expression of CRISPR-Cas systems and anti-CRISPR proteins in EC and KP strains under various infection microenvironments as well as the acquisition of carbapenemase-carrying plasmids.

**Figure 5:**
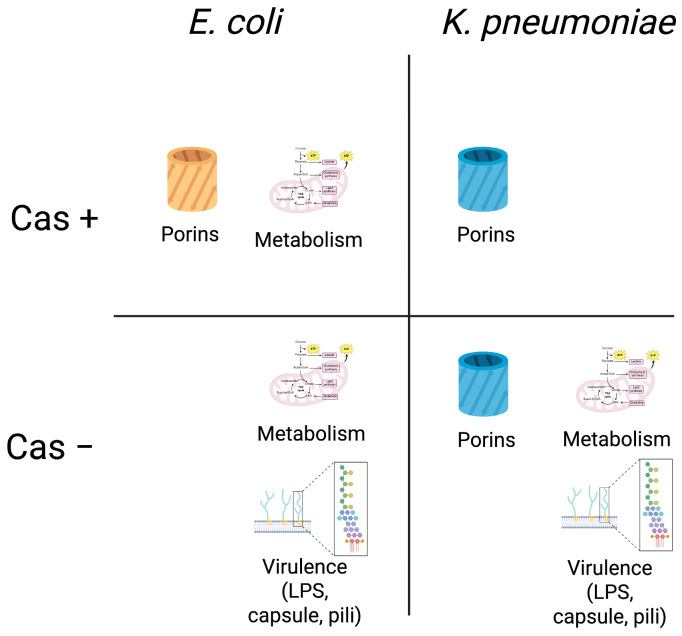
Summary of nonsynonymous mutations acquired in evolved EC and KP differentiated by the presence or absence of CRISPR-Cas system. Created in BioRender. Kalu, M. (2025). Images are representative of systems affected by nonsynonymous mutations found in evolved strains (see Table 2 and Table 3 for the specific genetic mutations identified). Cas −, absence of complete system; Cas +, presence of complete system.

Interestingly, we observed a difference in the acquisition of porin mutations between EC and KP pairs, with porin mutations mainly identified in CRISPR-Cas positive EC strains while present in KP strains irrespective of CRISPR-Cas systems (Figure 5). However, contrary to our observations, one study observed that porin mutations were acquired in ST111 KP strains when the CRISPR system was deleted, though this may be related to specific strain genetic background [7]. We also observed that porin mutations that resulted in termination of the protein contributed to large increases in meropenem MICs in KP strains compared to EC, regardless of the presence of CRISPR systems. We speculate that porin mutations may impart a lower fitness cost to KP than to EC, thereby representing a less stringent path towards evolving carbapenem resistance in ESBL-KP strains. It is possible that the acquisition of porin mutations is not significantly impacted by the presence and activity of CRISPR-Cas systems in KP strains given the propensity for genetic manipulation and diversity observed in global clones such as ST258.

Notably, mutations in virulence-associated genes were acquired mainly on the chromosome by CRISPR-Cas negative EC and KP strains. Most of the virulence genes were involved in capsule and LPS biosynthesis, potentially emphasizing the importance of these structures to biofilm formation, where plasmids can be exchanged (Figure 5). Several studies have shown the acquisition of resistance and virulence traits on plasmids; however, the rates of mutation or plasmid acquisition in CRISPR-Cas negative strains compared to CRISPR-Cas positive strains is largely unknown, even though active exclusion of plasmid acquisition by CRISPR-Cas systems has been described in other species [35,36,37,38,39].

Interestingly, metabolism-related genetic mutations were observed on the chromosome of the evolved EC strains irrespective of CRISPR-Cas background and carbapenem susceptibility and in CRISPR-Cas negative evolved KP strains that were also *blaKPC*-bearing CRKP (Figure 5). These observations suggest that the acquired mutations in ESBL-EC evolved strains may be an adaptation to support long-term colonization in the host, while acquired mutations in ESBL-KP evolved strains may support the acquisition of plasmid *blaKPC* conferring carbapenem resistance. Overall, evolved EC strains acquired fewer mutations in metabolic genes compared to those observed in KP strains. Most metabolic genes with nonsynonymous mutations encoded for transporters; *lacY*, lactose permease, and *dtpA* and *dtpC* encoding proteins involved in the transport of dipeptides and tripeptides. Peptide transporters have also been shown to be significantly downregulated upon plasmid acquisition [40]. DtpA specifically is controlled by osmolarity regulator OmpR and mutations in *dtpA* have been found in CRKP strains [8,41]. This is the first description of *dtpA* mutations in a carbapenem-heteroresistant EC strain. Among those mutations observed in the evolved KP strains, many were related to the electron transport chain and glucose metabolism. Specifically, mutations in *metF*, methylenetetrahydrofolate reductase, *gyrA*, and DNA topoisomerase subunit A were observed in a CRISPR-Cas positive, *blaKPC* negative KP strain which developed carbapenem resistance. Both genes have been linked to changes in antibiotic susceptibility. MetF is part of the methionine biosynthetic pathway and has been shown to be significantly downregulated upon acquisition of multidrug-resistant plasmid [40]. GyrA has been associated with the development of resistance to ciprofloxacin [42]. It is possible that the previous acquisition of ESBL-containing plasmids, in addition to antibiotic exposure, gave rise to these mutations.

The influence of metabolic gene mutations on antibiotic resistance has been previously demonstrated, with mutations in metabolic genes conferring protection of the bacterium against antibiotics [42,43,44]. Specifically, mutations in genes such as *sucA* encoding 2-oxoglutarate dehydrogenase conferred significant carbenicillin resistance in EC, and response regulator *qseB* involved in lipid A modifications imparts tolerance to polymyxin B, gentamicin, and amikacin in EC. One study also highlighted the accumulation of single nucleotide polymorphisms of groups of metabolic genes contributing to predicted antibiotic resistance phenotypes by machine-learning in EC, with meropenem resistance conferred through alterations in genes related to peptidoglycan metabolism (*murG*, *mraY*, and *glmM*), and lipopolysaccharide biosynthesis (*lptG*) [45]. We observed few mutations in lipopolysaccharide biosynthesis genes (*rfaQ* and *lptF*) in two ESBL-producing EC strains that developed carbapenem heteroresistance. Additionally, mutations in sensor kinase *cpxA* were observed linking resistance to imipenem in EC strains, while the two component system regulator unit CpxR/CpxA has been shown to control *blaKPC* expression and dissemination in KP [46,47]. Only one KP strain that developed full carbapenem resistance was observed to have both the *blaKPC* gene and mutation in *cpxA* in this study.

Several limitations of this study are noteworthy. First, all strains were collected from a single center; however, MLST analysis indicates that a wide genetic background was represented in our study strains. Second, evolved strains were selected from all recurrent infections or repeated colonization within two years. It is possible that with the inclusion of strains that have evolved over a longer period of time in humans, more apparent changes in resistance phenotypes can be observed. The sample size was limited for each group based on carbapenem susceptibility in the evolved strain and the number of strains in each group was not balanced between EC and KP as reflected by the frequency of resistance phenotypes encountered in the clinical setting. Future studies will characterize the resistance, virulence, and metabolic genes carried on plasmids acquired in EC and KP strains with and without CRISPR-Cas systems over time. Nonetheless, this study is the first to directly compare differences between ESBL-EC and -KP strains in their evolutionary paths towards increased carbapenem resistance by tracking the in-human evolution of clinical strains exhibiting different resistance phenotypes isolated from the same patient.

## 5. Conclusions

In summary, this study identified differential acquisition of plasmid-bearing *blaKPC* as well as chromosomal mutations in resistance, virulence, and metabolic genes between ESBL-EC and ESBL-KP strains evolving in humans under antibiotic pressure. We observed that strains belonging to either ST131 or ST258 were likely to carry plasmid-bearing *blaKPC* in the absence of CRISPR-Cas systems. Additionally, the prevalence of porin, virulence, and metabolism-related genetic mutations was differentially associated with the presence of CRISPR-Cas systems in evolved EC and KP strains. While carbapenem therapy is considered the treatment standard for serious infections caused by ESBL-producing *Enterobacterales,* irrespective of bacterial species, our findings brought to light the strain-specific responses in evolving carbapenem resistance among clinical EC and KP strains representing diverse genetic backgrounds. A corollary to our findings is that strain-specific antibiotic treatment strategies may need to be considered to disrupt the subsequent development of carbapenem resistance among *Enterobacterales* effectively. Future studies should investigate the differential role of antibiotic exposure in the strain-specific evolution of carbapenem resistance in ESBL-producing *Enterobacterales* to better inform antimicrobial stewardship efforts.

## Figures and Tables

**Table 1:** Partial loss of CRISPR-Cas systems identified in evolved strains.

Index Strain ^a,b^				Evolved Strain ^a,b^			
**Isolate** **ID**	**AMR ^c^**	**Cas**	CRISPR Spacers 5′-3′	Isolate ID	AMR	Cas	CRISPR Spacers 5′-3′
CK063	ESBL EC	Cas3	CGTTTTTAGCCTACCTATAAGGAATTGAAAC	CK178	ESBL EC	none	GCCGGATGCGGCGTGAACGCCTTATCCGGCCTACAAAAGAAATGCAG
			CCACCTTTTTTACCTGCTTCAGATGC				TTTTTGATAGTTGGAGTCGCTTTGTCTT
			ATCTGCCTGTACGGCAGTGAACT				TCTACAAGGACACAGACACACTTC
							ATCTGCCTGTACGGCAGTGAACT
PN312	ESBL EC	Cas3	**GCCGGATGCGGCGTGAACGCCTTATCCGGCCTACAAAAGAAATGCAG**	DS181	ESBL cHR EC	none	**GCCGGATGCGGCGTGAACGCCTTATCCGGCCTACAAAAGAAATGCAG**
			CGACCCCCACCATGTCAAGGTGGTGCTCTAACCAACTGAGCTA				CCAGAGAAGCCGCCAAAGCCGCTTCCGCC
			GTTTTTAGCCTACCTATAAGGAATTGAAACAGGT				TTTTTGATAGTTGGAGTCGCTTTGTCTT
			GTTTTTAGCCTACCTATAAGGAATTGAAAC				AGTTCACTGCCGTACAGGCAGCT
			CCACCTTTTTTACCTGCTTCAGATGC				
			ATCTGCCTGTACGGCAGTGAACT				
KT168	ESBL KP	Cas5	**TTGTGCCAACAGAATGCCAACAAAGTGCCA**	KT212	CRKP	none	**TTGTGCCAACAGAATGCCAACAAAGTGCCA**
			**AATAAAAACCATAAAAACCACAGT**				**AATAAAAACCATAAAAACCACAGT**
			GTTTTTAGCCTACCTATAAGGAATTGAAAC				
			AAGGCGTCAGCCGCCGCCCGGCA				

^a^ Conserved spacer sequences are bolded. ^b^ Partial loss is defined as loss of Cas protein-encoding gene, array, or both. ^c^ AMR; antimicrobial resistance; ESBL EC (*E. coli*), ESBL KP (*K. pneumoniae*), ESBL cHR EC (carbapenem-heteroresistant *E. coli*), CRKP (carbapenem-resistant *K. pneumoniae*). Conserved spacers between the index and evolved strain are bolded.

**Table 2:** Evolved EC strains containing nonsynonymous mutations in resistance, virulence, and metabolic genes.

Genes Mutated	Gene Function	Total N = 27	CRISPR-Cas Positive n = 6	CRISPR-Cas Negative n = 21	ESBL-ESBL n = 16	ESBL-cHR n = 10	CREC-CREC n = 1
**Resistance**							
*ompC*	porin OmpC	1 (3.70%)	1 (16.67%)		1 (6.25%)		
*ompF*	porin OmpF	1 (3.70%)	1 (16.67%)			1 (1.00%)	
*ompR*	Two component system response regulator OmpR	1 (3.70%)	1 (16.67%)			1 (1.00%)	
**Virulence**							
*rfaQ*	lipopolysaccharide core heptosyltransferase RfaQ	1 (3.70%)	1 (16.67%)			1 (1.00%)	
*lptF*	LPS export permease LptF	1 (3.70%)		1 (4.76%)		1 (1.00%)	
*wzzB*	LPS O-antigen chain length determinant protein WzzB	1 (3.70%)		1 (4.76%)	1 (6.25%)		
*arnD*	4-deoxy-4-formamido-L-arabinose- phosphoundecaprenol deformylase	1 (3.70%)		1 (4.76%)		1 (1.00%)	
*wcaM*	colanic acid biosynthesis protein WcaM	1 (3.70%)		1 (4.76%)	1 (6.25%)		
*waaU*	glycosyltransferase family 9 protein	1 (3.70%)		1 (4.76%)			1 (100%)
*fyuA*	siderophore yersiniabactin receptor FyuA	1 (3.70%)		1 (4.76%)		1 (1.00%)	
*entF*	enterobactin synthetase EntF	1 (3.70%)		1 (4.76%)	1 (6.25%)		
*ycgR*	flagellar brake protein	1 (3.70%)		1 (4.76%)		1 (1.00%)	
*fecR*	ferric citrate uptake regulator FecR	1 (3.70%)		1 (4.76%)	1 (6.25%)		
*agn43*	autotransporter adhesin Ag43	2 (7.40%)		2 (9.52%)	1 (6.25%)	1 (1.00%)	
*iutA*	ferric aerobactin receptor IutA	1 (3.70%)		1 (4.76%)		1 (1.00%)	
*yggR*	type IV pilus twitching motility protein PilT	1 (3.70%)		1 (4.76%)		1 (1.00%)	
*papX*	transcriptional regulator PapX	1 (3.70%)		1 (4.76%)		1 (1.00%)	
*cheY*	chemotaxis response regulator CheY	1 (3.70%)		1 (4.76%)		1 (1.00%)	
**Metabolism**							
*cydA*	cytochrome ubiquinol oxidase subunit I	1 (3.70%)	1 (16.67%)			1 (1.00%)	
*lacY*	lactose permease	1 (3.70%)		1 (4.76%)	1 (6.25%)		
*dtpA*	dipeptide/tripeptide permease DtpA	1 (3.70%)	1 (16.67%)			1 (1.00%)	
*dtpC*	dipeptide/tripeptide permease DtpC	1 (3.70%)		1 (4.76%)	1 (6.25%)		
*hycE*	formate hydrogenlyase subunit HycE	1 (3.70%)		1 (4.76%)	1 (6.25%)		
*fdrA*	acyl-CoA synthetase FdrA	1 (3.70%)		1 (4.76%)	1 (6.25%)		
*aaeB*	p-hydroxybenzoic acid efflux subunit AaeB	1 (3.70%)	1 (16.67%)			1 (1.00%)	

**Table 3:** Evolved KP strains containing nonsynonymous mutations in resistance, virulence, and metabolic genes.

Genes Mutated	Gene Function	Total N = 18	CRISPR-Cas Positive n = 5	CRISPR-Cas Negative n = 13	ESBL-ESBL n = 7	ESBL-CRKP n = 8	CRKP-CRKP n = 3
**Resistance**							
*ompK36*	porin OmpK36	2 (11.11%)	1 (20.00%)	1 (7.69%)		2 (25.00%)	
*ompK35*	Porin OmpK35	2 (11.11%)		2 (15.38%)		2 (25.00%)	
**Virulence**							
*ecpD*	fimbrial adhesin EcpD	1 (5.56%)		1 (7.69%)		1 (12.50%)	
*lptB*	Lipopolysaccharide export system ATP-binding protein LptB	1 (5.56%)		1 (7.69%)		1 (12.50%)	
*wzi*	capsule assembly Wzi family protein	1 (5.56%)		1 (7.69%)		1 (12.50%)	
*wbgU*	UDP-N-acetylglucosamine 4-epimerase	1 (5.56%)		1 (7.69%)			1 (33.33%)
**Metabolism**							
*rsxC*	electron transport complex subunit RsxC	1 (5.56%)	1 (20.00%)		1 (14.29%)		
*uhpT*	hexose-6-phosphate:phosphate antiporter	1 (5.56%)	1 (20.00%)		1 (14.29%)		
*kbl*	glycine C-acetyltransferase	1 (5.56%)	1 (20.00%)		1 (14.29%)		
*gyrA*	DNA topoisomerase subunit A	1 (5.56%)	1 (20.00%)			1 (12.50%)	
*metF*	methylenetetrahydrofolate reductase	1 (5.56%)	1 (20.00%)			1 (12.50%)	
*cpxA*	Sensor histidine kinase CpxA	1 (5.56%)		1 (7.69%)		1 (12.50%)	
*sodA*	superoxide dismutase	1 (5.56%)		1 (7.69%)		1 (12.50%)	
*cydB*	cytochrome d ubiquinol oxidase subunit II	1 (5.56%)		1 (7.69%)		1 (12.50%)	
*cyoA*	cytochrome o ubiquinol oxidase subunit II	1 (5.56%)		1 (7.69%)		1 (12.50%)	
*phoQ*	Sensor protein PhoQ	1 (5.56%)		1 (7.69%)			1 (33.33%)
*narL*	Nitrate/nitrite response regulator protein NarL	1 (5.56%)		1 (7.69%)			1 (33.33%)

**Table 4:** Minimum inhibitory concentrations of meropenem in EC and KP pairs with acquired porin mutations resulting in protein termination.

Isolate ID	MLST	AMR ^a^	Presence of CRISPR	KPC Gene	Porin Gene Mutated	Mutation	Meropenem MIC (μg/mL)
DS393	10	ESBL EC	Yes				≤0.015625
NB007		ESBL cHR EC	Yes		*ompF*	Addition; +GACC	0.03125
KT286	25	ESBL KP	No				0.0625
KT335		CRKP	No	No	*ompK35*	Addition; +T	8
AS071	307	ESBL KP	No				2
AS092		CRKP	No	No	*ompK35* *ompK36*	Addition; A(5)->(6) SNP; Q76 *	16
CK459	534	ESBL KP	Yes				0.03125
CK533		CRKP	Yes	No	*ompK36*	Deletion; Δ13 bp	8

^a^ AMR: antimicrobial resistance. Note: shaded row = evolved strain. (*) Denotes a stop codon.

## Data Availability

Whole genome sequence reads for this study are available as part of the National Institutes of Health National Center for Biotechnology Information Sequence Read Archive (NIH NCBI SRA) under project ID PRJNA1188153. All other data will be made available upon reasonable request to the corresponding author.

## Supplementary Materials

The following supporting information can be downloaded at: https://www.mdpi.com/article/10.3390/microorganisms13061387/s1. Supplemental Data S1, Excel file with nonsynonymous mutations identified between index and evolved EC and KP strains.

## Abbreviations

The following abbreviations are used in this manuscript:

EC	*Escherichia coli*
KP	*Klebsiella pneumoniae*
ESBL	extended-spectrum beta-lactamase
cHR	carbapenem heteroresistance
*blaKPC*	gene encoding *K. pneumoniae* carbapenemase enzyme
CRKP	Carbapenem-resistant *K. pneumoniae*
